# Perils of Total Colonic Aganglionosis Presenting in Neonatal Age

**Published:** 2014-07-10

**Authors:** Sarin YK, Raj P, Thakkar N

**Affiliations:** Department of Pediatric Surgery, Maulana Azad Medical College, New Delhi-110002, University of Delhi-110002

**Keywords:** Total colonic aganglionosis, Hirschsprung’s disease, Ileostomy, Kimura’s procedure

## Abstract

Background: The purpose of this study is to review the cases of total colonic aganglionosis seen in the span of ten years at a pediatric surgery unit of a tertiary care public hospital in New Delhi.

Methods: Medical records of twelve patients with total colonic aganglionosis were retrieved.

Results: Ten out of the twelve patients were males; seven were of the Muslim community. Average recorded birth weight was 2.2 kg. Ten patients presented with features of intestinal obstruction, while two presented with perforation peritonitis. Among the cases of obstruction, Hirschsprung's disease was suspected in eight cases (one was associated with Shah-Waardenburg syndrome), one case each was preoperatively diagnosed as ileal atresia and meconium ileus. Abdominal X-rays at presentation of all the neonates except in one with Shah-Waardenburg syndrome showed multiple air fluid levels. Contrast enema was done in five patients. It showed micro-colon in two patients, and typical question mark sign, dilated small bowel with transition zone in hepatic flexure and normal caliber colon in one each. All the patients underwent exploratory laparotomy. Intra-operatively, the transition zone was seen at distal ileum in ten cases and at hepatic flexure and transverse colon in one each. Biopsies of all the twelve patients eventually showed absence of ganglion cells in entire colon. Ileostomy was done in nine cases, colostomy in two and primary Kimura's procedure in one (this patient was discharged and lost to follow up). Left colonic patch with Swenson’s pull through with ileostomy was done for one patient on colostomy. His stoma was closed; he was eventually discharged and lost to follow up. In the other patient with colostomy, the stoma was closed and an ileostomy was created. Of all the patients on ileostomy, three expired in the immediate postoperative period. Four were lost to follow up. Two underwent Kimura's procedure; and expired few months later. One patient on ileostomy is awaiting further treatment.

Conclusion: The outcomes for total colonic aganglionosis in those presenting in neonatal age tend to be unsatisfactory in the developing countries.

## INTRODUCTION

Total colonic aganglionosis (TCA) is a rare form of Hirschsprung’s disease (HD) with incidence being 1 in 5,00,000 and it accounts for 5-10% of all the diagnosed cases of HD. High index of suspicion is required for the diagnosis of TCA. Mortality rate is high and most of them require long-term parenteral nutrition. Multiple operative procedures are mentioned for TCA, but there is no consensus regarding the superiority of one over the other. Most of the published literature of TCA has come from the developed world. We wish to share our experience to highlight the difficulties that we face in the developing world where the birth weights are low and the patients arrive late for management.

## MATERIALS AND METHODS

It is a retrospective study over a decade (2004-2014) of all the successive patients with histopathology-proven TCA who presented in neonatal age in one of the two units of the Department of Pediatric Surgery. Thirteen neonates of TCA were treated during this period. Records of one of the patients could not be retrieved; so 12 patients were evaluated for the study. During the same period, 39 neonates with HD but not having TCA were treated in the same Pediatric Surgical Unit. Further, 3 neonates during this period were operated with a pre-operative diagnosis of TCA, but were later found to have no aganglionosis. We evaluated the hospital records regarding the age, gender, weight at presentation, clinical presentation, antenatal history including consanguinity in marriage of parents, family history of affliction, pre-operative investigations including radiology, intra-operative diagnosis, extent of ileal involvement, surgical procedure(s), and outcomes in terms of post-operative morbidity and mortality and long-term follow up.

## RESULTS

Twelve neonates with TCA were managed between 2004-2014. Ten were males and two were females (M:F::6:1) (Table 1). Seven of the patients were from the Muslim community (58.3%) where consanguinity was frequently seen. Family history of HD was not elicited in any. Seven of these neonates were born to young primigravida mothers. Nine of them were delivered in hospitals (only one inborn) and other three were delivered at home. Birth weight was not recorded for these three home deliveries. Rest nine neonates had average birth weight of 2.2 kgs. Majority of the neonates presented with clinical features typical of HD; two patients presented with perforative peritonitis. The two late-presenters also had severe dyselectrolytemia at presentation. One patient had associated Shah-Waardenburg syndrome (SWS). All of these twelve patients had abdominal radiograph done at presentation, which showed multiple air-fluid levels except one with SWS. Contrast enema was done in five cases; two had micro-colon (Fig. 1), while one had transition zone reported at the level of hepatic flexure and one with normal caliber colon (SWS). Only one of the patients had typical question mark appearance on contrast enema. Pre-operative diagnosis of HD was considered in 8 cases; ileal atresia and meconium ileus were considered in one each. Two patients had perforative peritonitis, where preoperative diagnosis of necrotizing enterocolitis was entertained. All the patients eventually underwent exploratory laparotomy after initial stabilization. Intra-operatively, transition zone was seen in ileum in ten cases (Fig. 2). In two other patients, the operating surgeon recorded it at the level of hepatic flexure and transverse colon. Exact length of ileal involvement was not mentioned in the operative details of all the patients. Leveling ileostomy was done in nine cases and colostomy in other two; multiple seromuscular colonic biopsies and appendectomies were undertaken in all the patients. One patient underwent definitive surgery (extended Kimura's procedure with end ileostomy) as primary procedure; this patient was discharged and lost to follow up. All of them, including the two patients in whom there was transition zone in colon, showed absence of ganglion cells in the entire colon and appendix. In one of these two patients, the initial colostomy was closed and ileostomy was done. One patient with ascending colostomy underwent left colonic patch with Swenson's pull through with ileostomy. His ileostomy was closed at a later date. He was discharged and lost to follow up thereafter. 

**Figure:**
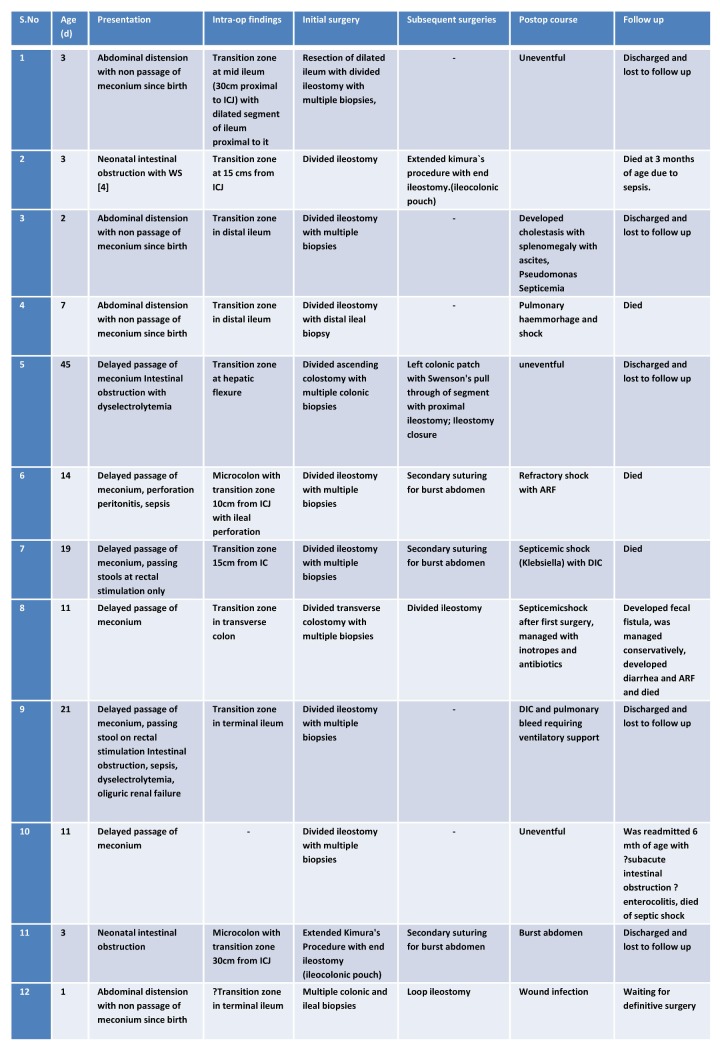
Table 1: Patient characteristics, surgical details and the outcomes.

**Figure:**
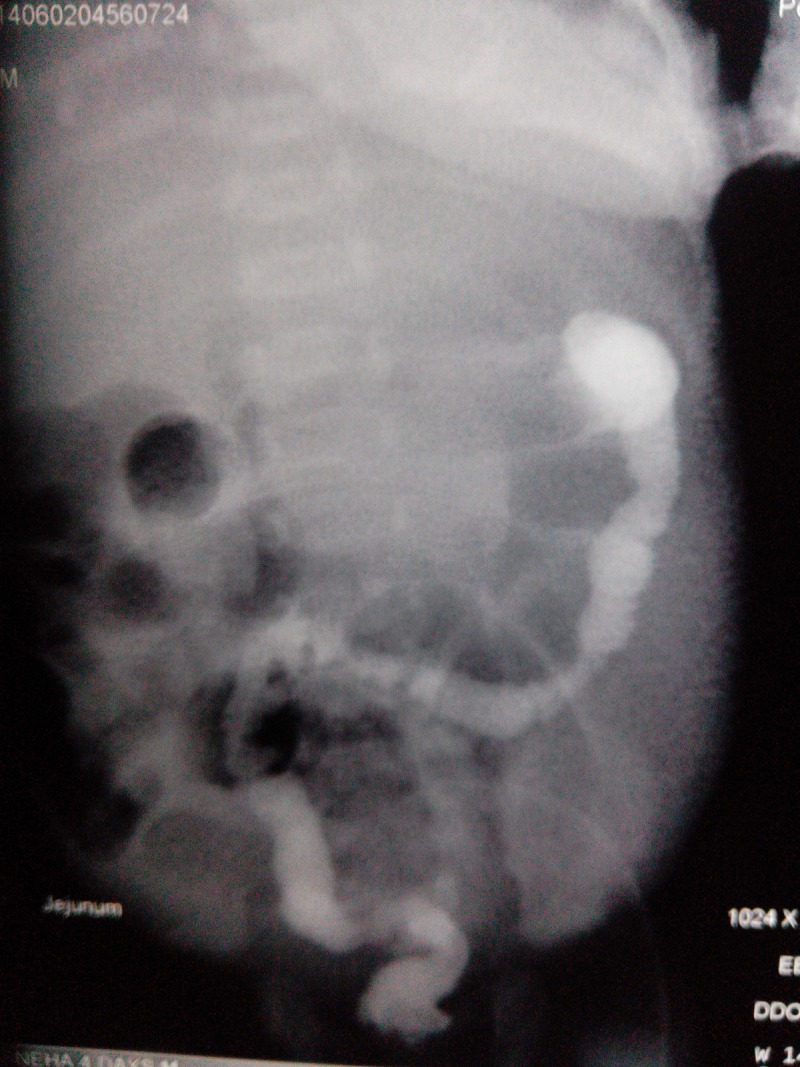
Figure 1: Contrast enema showing micro-colon.

**Figure:**
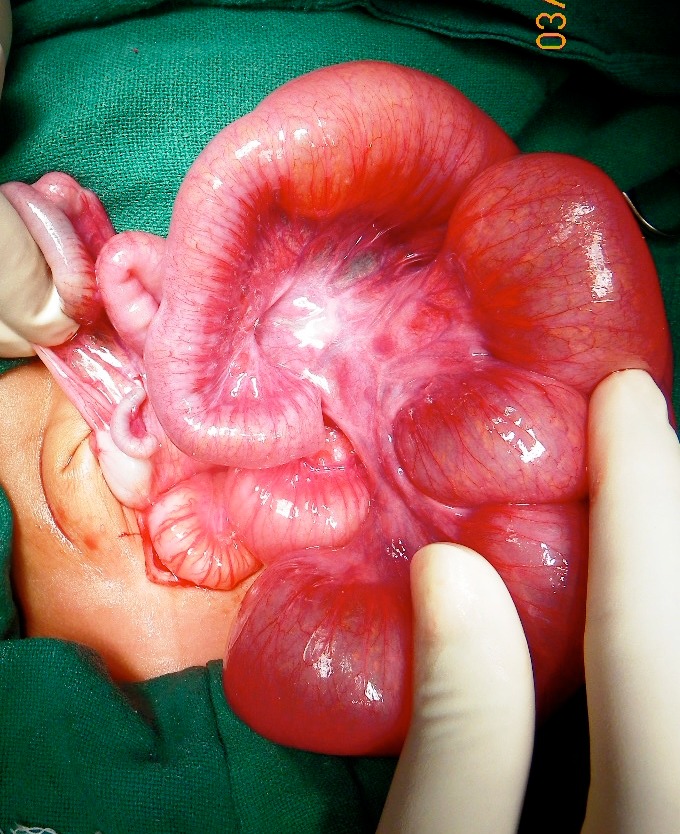
Figure 2: Intra-operative picture showing transition zone of aganglionosis in terminal ileum.

Three of the patients with ileostomy died in the immediate post-operative period due to sepsis. Four patients on ileostomy were lost to follow up and never turned up for definitive procedure. Two patients had definitive surgery in form of Kimura's procedure. Both succumbed to sepsis 3 months and 6 months respectively. One patient with ileostomy is awaiting definitive surgery.

## DISCUSSION

Total colonic aganglionosis is a rare disease with an incidence of 1 in 5,00,000 live births [1]. Due to its rarity, it is still a diagnostic and therapeutic challenge for pediatric surgeons worldwide. It is a severe form of Hirschsprung’s disease, occurring in 5-15% of the cases [2]. Zueler and Wilson first reported it in detail in 1948 [3].There is very little literature about this entity from the developing countries like India, probably because of the scarcity of cases and the difficulty in maintaining a long-term follow up among the poor, uneducated strata of patients.

Male to female ratio in our study was 6:1. This is more than that reported by Bischoff et al (2.47:1) [4] and Tabari et al (all females) [5]. This skewed ratio could possibly be due to the traditional and cultural beliefs in India where the female child is often unwanted and neglected, and thus not brought to the attention of healthcare facilities.

We found the disease to be more common among patients of the Muslim community (58.3%). This could be either related to the higher proportion of this community in the drainage area of our hospital, or it may be due to consanguinity in marriages; the latter needs to be explored with genetic studies and more research. We were unable to find any data in literature related to the religious background of the patients. There was no significant family history in our patients, unlike the study by Louw JH [6]. 

Seven neonates (63.6%) were born to young primigravida mothers. Average birth weight was 2.2kg. Age at presentation varied from 1 to 25 days, similar to other studies [6].

Ten patients (83.3%) presented with features of intestinal obstruction, of which eight had typical features suggestive of HD. The two late presenters had severe dyselectrolytemia that could be easily explained. Two (16.66%) presented with perforative peritonitis. These results vary from a study by Menezes M et al [8], where patients also presented with enterocolitis and severe constipation. However in their study, approximately one-third of the patients had presented after the neonatal period. It is worth mentioning that some of them could be detected quite late in childhood [7]. Of the eight patients with typical features of HD, one was associated with Shah-Waardenburg syndrome. This association has been previously reported by the first author (S. No. 2 in Table 1) [9].

Total colonic aganglionosis is seldom diagnosed pre-operatively. This fact highlights the need for having a very high index of suspicion for this condition. If this condition is suspected pre-operatively, frozen section biopsies should be ideally done to assess the level of aganglionosis in order to determine the level of diverting stoma, as many times clear transition zone could not be visible in TCA. This may lead to a wrong placement of stoma in an aganglionic segment, which is a very common operative error, and it occurred in two of our patients also. A definitive surgery can also be done at the first go, as was done in one of our patients. Unlike Menezes et al [8] and Escobar MA et al [10], we did not find any patient having a level of aganglionosis higher than the terminal ileum.

A number of surgeries have been mentioned in literature including Swenson, Martin, Duhamel, Martin modification of Duhamel, Kimura, endorectal pull through, Rehbein and direct ileo-rectal anastomosis for TCA, but none of them is universally accepted. Martin modification of Duhamel’s procedure and Kimura procedure are based on the following principle - to take advantage of the normal ganglionated small bowel for motility and aganglionated large bowel for water absorption, thus having an advantage of lesser number of stools a day, which are solid in consistency. However, even these two procedures are not free of complications like fecal stasis and increased incidence of enterocolitis. Direct ileo-rectal anastomosis though free of enterocolitis suffers from other complications like frequency of stool, perianal excoriation, stricture and fistula formation.

Eleven out of the twelve patients underwent an initial diverting enterostomy, similar to other studies [10, 11].

The mortality rate is in the series was 41.6%, however the actual rate may be higher as there may be hidden mortalities amongst the patients that were lost to follow up. This rate is much higher than that reported from the western world [6, 8, 11]. The dismal outcomes reflect on the inadequacy of health services as well as the attitudes, poverty and illiteracy prevailing in the families of these neonates. Abandonment of treatment is very commonly noted in the developing world for all the neonatal surgical conditions that need multiple surgeries. Performing single-stage surgical management could obviate this and we need to strive to evolve such a procedure in the future.

## Conclusion

We conclude that the outcomes for total colonic aganglionosis in those presenting in neonatal age tend to be unsatisfactory in the developing countries. The parents need to be counseled well for the long follow-ups that are mandated in these patients. A national registry is necessary for better auditing if we wish to improve our long-term outcomes. 

## Footnotes

**Source of Support:** Nil

**Conflict of Interest:** The author is editor of the journal but the manuscript is handled independently by other editors and he is not involved in decision making of the manuscript.

